# Consanguinity, inbreeding coefficient, fertility and birth-outcome in population of Okara district, Pakistan

**DOI:** 10.12669/pjms.37.3.2263

**Published:** 2021

**Authors:** Aqeela Nawaz, Muhammad Zaman, Sajid Malik

**Affiliations:** 1 Aqeela Nawaz, M.Phil. Human Genetics Program, Department of Zoology, Quaid-i-Azam University, Islamabad, Pakistan; 2 Muhammad Zaman, PhD. Department of Sociology, Quaid-i-Azam University, Islamabad, Pakistan; 3 Sajid Malik, PhD. Human Genetics Program, Department of Zoology, Quaid-i-Azam University, Islamabad, Pakistan

**Keywords:** Epidemiology, Demography, Consanguineous unions, Fertility outcome, Reproductive outcome, Sex ratio, Child morality, Child morbidity

## Abstract

**Objectives:**

This study was aimed to illustrate the determents of consanguinity and inbreeding coefficient-F (ICF) in the population of Okara district of Pakistan and to elucidate the impact of consanguinity on fertility and birth outcome.

**Methods:**

Through a cross-sectional sampling design, 1,521 married women were recruited from Okara district during 2016-2017. Data on demographic variables, marital union types, subject’s fertility, and reproductive outcome, were gathered in face-to-face interviews. Descriptive statistics and multivariable logistic regression were employed.

**Results:**

The prevalence of consanguineous unions (CU) was calculated to be 61% yielding ICF=0.0356. Multivariable regression analyses revealed that six variables including younger age at marriage, joint family structure, caste-system of spouse, exchange marriage, matrimonial distance, and parental consanguinity, were significant predictors of consanguinity. The women having CU had significantly higher mean fertility, mean live-births and mean live-born sons compared with subjects having non-consanguineous unions (NCU). However, there were no significant differences in the average mortalities, i.e., prenatal, postnatal and <5 years, between the mothers with CU and NCU.

**Conclusion:**

The prevalence of consanguineous unions (CU) in Okara district is quite high like other inbred populations of Pakistan. The striking findings of this study are the higher mean fertility and mean live-births in women with CU. The likely reasons underlying this phenomenon have been discussed.

## INTRODUCTION

Pakistan is an interesting ground for the study of various aspects of demography like assortative mating and consanguinity.^1^ The pattern of marital alignments and consanguinity is useful in elucidating the socio-biological structure of populations and assessing health and disease patterns.^2^,^3^ Elevated consanguinity has been reported to be associated with higher incidences of congenital anomalies, child mortality and adult morbidity, and decreased fertility.^3^-^6^

Consanguinity is a deeply rooted social and cultural trend in Pakistan.^7^ The major cause for an advantage of consanguineous unions (CU) is socio-cultural instead of economic benefits. A review of the literature on this subject revealed that despite certain generalizations the factors underlying consanguinity vary in different populations of Pakistan.^2^,^7^-^9^

The world population has been divided into four broad categories based on the prevalence of CU, i.e., <1%, 1-10%, 10-50%, and unknown.^5^ Pakistan falls into the third category where an overall high prevalence of consanguinity has been reported.^5^,^10^,^11^ Here, consanguinity has been studied primarily in upper Punjab and few populations of Khyber Pakhtunkhwa province.^2^,^8^,^9^,^11^ The pattern of consanguinity remains unknown for most of the Southern and Western regions which may comprise populations with distinct breeding structures. Hence, the categorization of Pakistani populations on the basis of consanguinity level is not yet possible. To this end, this study was aimed at the determination of consanguinity, fertility and birth-outcome in the population of Okara district of Punjab, Pakistan.

## METHODS

### Study Population:

Okara district is situated in the South-East of Punjab, Pakistan. According to the 2017 census, the district’s population was three million and >80% was rural. The district comprises three tehsils namely Okara, Depalpur and Renala Khurd.^12^ The most prominent caste-systems are Arain, Bhatti, Jutt, Kharal, Khokhar/Malik, Kamyanay, Muslim-Rajput, and Rajput.

### Sampling Strategy and Definitions:

Through a cross-sectional sampling design, ever-married females were enrolled during 2016-2017. There were 19 different sampling sites encompassing main towns/villages of the district. The fieldwork was coordinated by the lady-health-visitors and paramedical staff. This study was approved by the ethical review committee of Quaid-i-Azam University (DAS/13-651; June 3, 2013).

After a verbal consent approval, the data were collected on a structured proforma. Information on marital union types and demographic/household parameters (including tehsil, rural/urban residence, subject/spouse age, subject/spouse literacy, occupation of subject/spouse, family structure, household type, exchange marriage, subject’s age at marriage, matrimonial distance, and parental consanguinity) was collected (as described elsewhere).^2^,^7^,^8^ Inbreeding coefficient-F (ICF) was calculated from the proportion of CU types in total marriages in a respective category.^5^,^7^ Data were also gathered on fertility, gap in the first pregnancy, live-births, and reproductive loss, i.e., neonatal, postnatal and ≤5 years mortality. Descriptive summaries were generated and the significance of deviation from random distribution was checked at p<0.05. Multivariable analyses were carried out through logistic regression, performed in various tiers where consanguinity was put as a dichotomous variable and the dependent variables were included in the model step-by-step.

## RESULTS

A total of 1,521 married women ranging in age from 15-to-80 years were enrolled. A total of 933 (61.3%) subjects had CU, yielding ICF=0.0356 (Table-I). First cousin (FC) unions were the highest in proportion and accounted for 50.2% (n=763) of the total sample. Double first cousin, first-cousin-once-removed, and second cousin marriages were 1.4%, 6.6%, and 3.2%, respectively. The unions among second-cousin-once-removed, distantly related and non-related were 0.1%, 28.1% and 10.4%, respectively.

**Table-I:** Consanguineous unions and inbreeding coefficient with respect to demographic variable.

Variable*	Consanguineous unions, No. (%)	Total marriage, No.	Odds ratio#	Inbreeding coefficient (ICF)
***Rural/urban residence***				
Rural	444 (65.5)	678	1.37	0.0385
Urban	489 (58.0)	843	Reference	0.0333
Total	933 (61.3)	1,521		0.0356
***Literacy (subject)***				
Illiterate	504 (64.9)	776	1.13	0.0379
Literate	429 (57.6)	745	Reference	0.0333
***Literacy (spouse)***				
Illiterate	351 (65.2)	538	1.11	0.0383
Literate	582 (59.2)	983	Reference	0.0342
***Occupational categories (subject)***				
House-wife	848 (62.6)	1355	1.59	0.0364
Working women	85 (51.2)	166	Reference	0.0289
***Occupational categories of spouse (spouse)***				
Manual jobs	143 (66.5)	215	1.88	0.0392
Agriculture	414 (63.6)	651	1.65	0.0370
Sales/business	167 (62.1)	269	1.55	0.0360
Professional	67 (53.2)	126	1.07	0.0298
Others	83 (57.2)	145	1.27	0.0330
Services	59 (51.3)	115	Reference	0.0303
***Caste-system (spouse)***				
Kharal	36 (70.6)	51	1.43	0.0426
Muslim-Rajput	48 (69.6)	69	1.41	0.0387
Jutt	46 (67.6)	68	1.37	0.0345
Khokhar/Malik	87 (65.9)	132	1.34	0.0405
Arain	179 (62.2)	288	1.26	0.0368
Bhatti	100 (60.6)	165	1.23	0.0346
Rajput	89 (57.1)	156	1.16	0.0344
Others	315 (60.0)	525	1.22	0.0345
Kamyanay	33 (49.3)	67	Reference	0.0287
***Family structure***				
Joint family	528 (68.1)	775	1.80	0.0401
Nuclear family	405 (54.3)	746	Reference	0.0310
***Household type***				
Paternal	830 (62.8)	1,321	1.59	0.0365
Maternal	103 (51.5)	200	Reference	0.0301
***Exchange marriage***				
Yes	167 (78.0)	214	2.51	0.0467
No	766 (58.6)	1307	Reference	0.0338
***Subject’s age at marriage (years)***				
Up to 20	315 (56.5)	558	1.38	0.0337
>20	618 (64.2)	963	Reference	0.0368
***Matrimonial distance (km)***				
Up to 5	609 (68.6)	888	1.87	0.0405
>5	304 (53.8)	565	Reference	0.0304
***Parental consanguinity***				
Yes	272 (73.5)	370	1.76	0.0428
No	214 (61.1)	350	Reference	0.0346

* only statistically significant variables are reported

# estimated through single factor logistic regression

Contingency test statistics showed that the CU were significantly associated with variables like rural/urban residence (higher in subjects from rural background; OR: 1.37), literacy of subjects and spouses (higher in illiterates; OR: 1.13 and 1.11, respectively), occupational status of subjects and spouses, joint family structure (OR: 1.80), household type (higher in paternal type; OR: 1.59), exchange marriage (OR: 2.51), age at marriage (higher in younger age; OR: 1.38), matrimonial distance (higher in less distance; OR: 1.87), and parental consanguinity (OR: 1.76). The differences in the distribution of CU and NCU appeared statistically not significant with respect to tehsils, mother tongue, caste-system of subjects/spouses, current age of subject/spouse, and marriage year (data not shown).

In logistic regression, six variables emerged as significant predictors of consanguinity, i.e., caste-system of spouse, age at marriage, exchange marriage, matrimonial distance, family type, and parental consanguinity, while the overall model was highly significant (Table-II). Temporal analyses across 10 years intervals revealed that the rate of consanguinity fluctuated between 59%-to-63% and any decreasing or increasing trend over the years was not conspicuous (Chi-test for trend; p=0.627).

**Table-II:** Significant predictors of consanguinity as depicted by multivariable logistic regression.

Variables in final model	Odds ratio	St. Err.	p-value	95% CI
Age at marriage (subject)	1.65	0.29	0.004	1.17-2.33
Caste-system (spouse)	1.10	0.05	0.049	1.00-1.21
Family structure (joint)	1.62	0.28	0.005	1.16-2.27
Matrimonial distance (up to 5 km)	1.77	0.31	0.001	1.25-2.50
Parental consanguinity (yes)	1.71	0.29	0.002	1.23-2.39
Exchange marriage (yes)	2.42	0.65	0.001	1.43-4.09
_cons	0.02	0.01	0.0001	0.00-0.08

Ever pregnant women were 1,958 (90.9%) (Table-III). The subjects having CU had significantly higher mean fertility than subjects having NCU (4.08±2.98 vs. 3.74±2.81, respectively; p=0.007). A proportionately higher number of women with CU had a gap of >24 months in their first pregnancies compared to women with NCU (p=0.03). Among the ever-pregnant women, mean live-births were significantly higher in women who had CU compared with the subjects having NCU (3.62±2.68 vs. 3.30±2.50, respectively; p=0.006). The differences were also statistically significant in the case of mean live-born sons (1.90±1.64 vs. 1.64±1.47, respectively; p=0.0002), but not in live-born daughters (p=0.406). Further, there were statistically no significant differences between the mothers with CU and NCU with respect to average mortalities (i.e., prenatal, postnatal and total).

**Table-III:** Subject fertility and live-births in consanguineous and non-consanguineous unions.

Parameter	Consanguineous unions	Non-consanguineous unions	Total	p-value*
Average age (yrs)	34.99±13.36	35.16±13.31	35.06±13.34	0.67
***Fertility***				
Ever pregnant women, No. (%)	872 (60.8)	562 (39.2)	1,434 (94.3)	
Fertility: pregnancy/women (mean±SD)	4.08±2.98	3.74±2.81	3.94±2.91	0.007
***Gap in first pregnancy***				
Up-to 24 months (%)	85.8	89.7	87.3	0.03
>24 months (%)	14.2	10.3	12.7	
***Live-births***				
Total live-births (No.)	4,479	3,024	7,503	
Live-births/women (mean±SD)	3.62±2.68	3.30±2.50	3.48±2.61	0.006
Live-birth: sons (mean±SD)	1.90±1.64	1.64±1.47	1.79±1.58	0.0002
Live-birth: daughters (mean±SD)	1.72±1.61	1.66±1.59	1.69±1.60	0.406
***Mortalities***				
Data available on mothers (No.)	1,239	915	2,154	
Mortality/women (mean±SD)	0.47±1.11	0.44±1.17	0.45±1.14	0.528
Prenatal mortality (mean±SD)	0.26±0.85	0.27±0.93	0.26±0.88	0.658
Postnatal mortality (mean±SD)	0.21±0.73	0.16±0.74	0.19±0.73	0.169
***Child morbidity (≤5 years)***				
Mortality in sons, No. (%)	43 (3.8)	36 (4.4)	79	OR=0.86
Mortality in daughters, No. (%)	41 (3.6)	24 (2.9)	65	OR=1.23

* (student T-test; statistical findings/significance did not differ when analyses were repeated through Mann-Whitney test and unpaired t-test with Welch’s correction).

## DISCUSSION

Consanguinity was estimated to be 61.3% in the overall sample of Okara population, and the corresponding ICF was 0.0356 which is comparable to other populations of Pakistan; for instance, 0.0355 in RahimYar Khan and 0.0348 in Sargodha, and 0.0348 in Bhimber, AzadJammu Kashmir.^2^,^7^,^13^ Further analyses revealed that intra-caste marriages were 90% in the total unions while their proportion ranged from 96% in Arain to 77% in Mirza caste-systems (data not shown). Although Okara is close to the metropolitan region of Lahore yet it has high level of consanguinity. This reflects that greater metropolitan regions are not exception of the CU. People remain intact to their cultural values rather than accepting the norms and values of the metropolitan culture which is reported to have low consanguinity.^14^^,^

Multivariable analyses revealed that six variables were the significant predictors of consanguinity. For instance, with respect to family structure, the subject belonging to joint/extended family type had the higher occurrence of consanguinity. In joint family setup, CU are preferred because of the compatibility between subfamilies, i.e., similar tradition, education and economic levels, and among cousins living in the same environment.^2^,^7^ Consanguinity helps them to settle minor domestic conflicts and social and economic problems.^14^ Further, the rate of CU was significantly higher in subjects who had younger age at marriage. The analyses demonstrated that rate of consanguinity gradually declined as the age at marriage delayed. In joint family system, a large number of siblings reached to marriageable age.^3^,^7^,^14^ The parents prefer to marry their daughters among the relatives at her younger age because relative gives more respect to their daughters at a younger age.^13^-^15^ In economically better-off families particularly in urban populations, the late age marriages may be commenced due to the time spent in completing education and career development which leads to a low choice of mates among the close-kins, thus resulting in outbreeding.^5^,^15^

The exchange marriages were observed to be positively associated with consanguinity both in univariate and multivariable analyses (OR: 1.33 and 2.62, respectively). Exchange marriages are customarily practiced in the rural areas of Punjab and Sindh provinces.^14^,^16^ Then, parental consanguinity was also observed to be a significant predictor of subject’s consanguinity in the present study. This observation supports the notion that consanguinity is a family tradition that is practiced generations after generations.^14^,^17^

Our analyses showed that the average fertility was higher in women with CU compared to the women with NCU. Further, the average live-births per woman were also significantly higher in subjects who had CU. Interestingly, a similar trend was also witnessed in RahimYar Khan, Pakistan, and several other populations of Asia and Africa.^2^,^5^ This phenomenon could be explained by the fact that owing to the marriages at younger age in CU, the first birth occurs at an earlier age and the reproductive and fertile period of the women remains significantly longer.^5^ Further, several other fertility determinants are potentially confounding with CU which include, but not limited, to the duration of the marriage, low socio-economic status, rural residence and lower contraceptive use.^4^,^18^ However, 14% mothers with CU had a gap of >24 months in their first pregnancies compared to 10% mothers with NCU. This observation needs further investigations in extended data sets from other populations.

Nonetheless, the average male live-births were significantly higher in women with CU compared with the women with NCU (Table-III). The difference was, however, not significant in average female live-births. It has been previously suggested that consanguinity may affect the secondary sex ratio and the sex ratio decreases as consanguinity increases.^19^ However, sex ratio is also confounded by other direct or indirect variables like early age at marriage, paternal age, firstborn proportion and mother’s age.^20^ Further studies are warranted to understand this phenomenon in cosmopolitan populations.

### Strength and Limitaitons of the study:

The strength of the present study are: first-hand data, large sample size, and coverage of large number of demographic variables. The potential limitations of this study could be that the high representation of subjects from urban areas and self-reported data on reproductive health. Further, this study does not document stillbirths, congenital anomalies, and maternal morbidity.

## CONCLUSION

The overall rate of CU in Okara district is high like other inbred Pakistani populations. Here, higher mean fertility and mean live-births in subjects with CU are interesting findings and need further studies.

### Authors Contribution:

**SM:** Conceived, designed and supervised the study; statistical analysis and manuscript writing

**AN:** Data collection and manuscript writing

**MZ & SM:** Edited, reviewed and approval manuscript

**SM** is responsible and accountable for the accuracy and integrity of data.
